# Up-Front and Salvage Transoral Laser Microsurgery for Early Glottic Squamous Cell Carcinoma: A Single Centre Retrospective Case Series

**DOI:** 10.3389/fonc.2018.00186

**Published:** 2018-05-28

**Authors:** Jeroen Meulemans, Jacqueline Bijnens, Pierre Delaere, Vincent Vander Poorten

**Affiliations:** ^1^Otorhinolaryngology-Head and Neck Surgery, University Hospital Leuven, Leuven, Belgium; ^2^Department of Oncology, Section Head and Neck Oncology, KU Leuven, Leuven, Belgium

**Keywords:** conservation surgery, laryngeal cancer, salvage surgery, transoral laser microsurgery, squamous cell carcinoma

## Abstract

**Introduction/aim:**

Transoral laser microsurgery (TLM) is a minimally invasive surgical alternative for radiotherapy (RT) in the primary management of early glottic cancer. More recently, TLM emerged also as a possible salvage treatment for selected radiorecurrent cancers. We reviewed outcomes of primary and salvage TLM performed in a Belgian tertiary referral center.

**Patients and methods:**

A retrospective review of records from 142 consecutive patients who underwent TLM was performed. Oncologic outcomes were evaluated by means of descriptive statistics and Kaplan–Meier estimates. Variation of estimated outcomes between different subgroups was evaluated using Log-Rank analysis.

**Results:**

Of 142 patients, 109 (76.8%) underwent TLM as a primary treatment and 33 (23.2%) were treated in a salvage setting for recurrent or second primary glottic cancer. cT classification in the up-front TLM group was cT1a in 72 (66.1%), cT1b in 11 (10.1%), and cT2 in 26 (23.9%) patients. In the salvage group, patients were cT/rT classified as cT1a–rT1a in 17 (51.5%), cT1b–rT1b in 1 (3.0%), cT2–rT2 in 14 (42.4%), and cT3–rT3 in 1 (3.0%) patients. All patients were cN0. Second-look TLM was performed in 28 patients (19.7%), and RT was associated as adjuvant therapy in 5 patients (3.5%). Mean follow-up was 51.6 months (SD = 38.4 months). Three-year overall survival (OS) was 94.1% (SE = 2.2%), 3-year disease-specific survival (DSS) 100%, 3-year disease-free survival (DFS) 80.1% (SE = 3.8%), 3-year local recurrence-free survival (RFS) 81.0% (SE = 3.7%), and 3-year ultimate local control rate with laser alone 89.2% (SE = 3.0%). Upon subgroup analysis, no differences in OS, DSS, and DFS were observed between the up-front and salvage group (log rank; *p* = 0.306, *p* = 0.298, and *p* = 0.061 respectively). However, local RFS and ultimate local control rate with laser alone were significantly higher in the primary treated TLM group (log rank, *p* = 0.014 and *p* = 0.012). Five-year laryngeal preservation rate was 89.7% (SE = 3.5%) in the total population, 100% in the upfront group, and 64.9% (SE = 9.8%) in the salvage group, a difference which proved statistically significant (Log-Rank, *p* < 0.001).

**Conclusion:**

This retrospective study confirms excellent oncologic outcomes of up-front TLM for early glottic cancer. In the salvage setting, TLM allows avoidance of total laryngectomy in the majority of cases.

## Introduction

In the US and North-western European countries, early (T1–T2) glottic squamous cell carcinoma (SCC) has traditionally been treated most commonly with primary external beam radiotherapy (RT), yielding excellent functional and oncologic results. The reported rates of local control with RT alone for T1 glottic SCC range from 84 to 95% (1). For T2 glottic tumors, local control rates between 50 and 85% have been reported (1). However, transoral laser microsurgery (TLM) emerged as a surgical alternative to RT for the primary management of early glottic SCC. TLM was introduced as a minimally invasive therapeutic technique mounting a CO_2_ laser on an operating microscope for treatment of laryngeal lesions by Strong and Jako (2). In Europe, TLM for the treatment of glottic malignancies was initially mainly propagated by Steiner in the 1980s (3), who expanded the indications of TLM to all tumor categories of the upper aerodigestive tract (4). The main feature of TLM is the concept of tumor adapted resection: the tumor is transected and removed piecemeal through the laryngoscope. Transection of the tumor reveals the depth of tumor invasion and allows for clear visualization of tumor margins, resecting the tumor with an adequate margin while leaving as much healthy tissue as possible, the anatomy of the organ being less disturbed as compared with open transcervical/translaryngeal approaches. This minimizes the adverse functional impact, while leaving all salvage options open, including (chemo)radiotherapy or radical surgery. Nowadays, TLM has a proven track record in the primary management of glottic cancer, combining local control rates comparable to primary irradiation with excellent laryngeal preservation rates (1, 5–9). More recently, TLM emerged as a possible salvage treatment for selected radiorecurrent laryngeal cancers (10–12). In this study, we review the outcomes of primary and salvage TLM performed in a Belgian tertiary referral center.

## Patients and Methods

### Patients

A retrospective study was conducted at an academic tertiary referral hospital (Department of Otorhinolaryngology, Head and Neck Surgery, University Hospitals Leuven, Leuven, Belgium). This study was approved by and carried out in accordance with the recommendations of the Institutional Review Board (University Hospital Leuven Committee for Medical Ethics). Informed consent for retrospective studies with anonymized data is not required according to Belgian law. The records of all patients who were scheduled for primary or salvage TLM for suspected glottic malignancy between 1999 and 2016 were retrospectively reviewed and analyzed. Exclusion criteria were as follows: definitive pathology other than SCC (benign lesions, plasmacytoma, verrucous carcinoma, etc.) and TLM performed by a surgeon other than the senior surgeon (Vincent Vander Poorten). The selection of patients with primary early glottic SCC for TLM was based on a combination of (1) an estimate of tumor extent based on laryngoscopic and CT-graphic evaluation so that tumor resection would be possible with relatively low impact on the patient’s voice and (2) patient’s preference following this advice. The ultimate decision to submit a patient with glottic SCC to TLM rather than RT always resulted from a multidisciplinary tumor board discussion during which the patient’s preferences were respected. As most patients were referred to our tertiary center following CT scanning and direct laryngoscopy and biopsy in a regional hospital, tumor board discussion was based on this referral information combined with thorough in-office laryngoscopy. If, based on this information, TLM seemed an adequate treatment option, a general tumor board agreement allows the surgeon to schedule a direct laryngoscopy and immediately proceed to TLM if an adequate resection is feasible. As such, procedures were partly planned as diagnostic laryngoscopy “with reserve.” This means that already preoperatively some doubt about the feasibility of TLM existed, and the patient was clearly informed that the peroperative decision to proceed to therapeutic TLM would depend on adequate exposure, adequate resection deemed possible and an expected acceptable voice function. When these preconditions were peroperatively not fulfilled, the procedure remained a pure diagnostic laryngoscopy with biopsy retrieval and/or debulking and RT was offered as a therapeutic alternative. Before surgery, patients were staged and screened for synchronous upper aerodigestive tract tumors and distant disease using esophagogastroduodenoscopy, chest X-ray, and ultrasound of the liver or CT chest abdomen, according to our general work-up protocol for patients with head and neck cancer. T-status was determined according to the International Union for Cancer Control (UICC) 7th edition staging system for malignant head and neck tumors (13).

### Treatment

All TLM procedures were performed by the same senior surgeon (Vincent Vander Poorten). Patients were under general anesthesia and ventilated using a small diameter endotracheal tube (5.5 or 6 mm) or jet ventilation. Different closed laryngoscopes (Karl Storz, Tuttlingen, Germany) were used to achieve optimal exposure of the glottic area. Before the actual TLM procedure, the extent and location of the tumor was assessed using 30° and 70° endoscopes. TLM was performed using a CO_2_ laser (Model 40 C, Sharplan, Israël, later on AcuPulse Duo, Lumenis, Israël) equipped with a micromanipulator attached to the operating microscope (OPMI Vario, Zeiss, Göttingen, Germany). Resections were performed according to techniques described by Steiner (3, 4). Except from the smallest glottic lesions which were removed en bloc, a piecemeal resection was achieved with cutting through the tumor, allowing peroperative exploration of depth of invasion, taking a precise extra margin, while preserving as much healthy tissue as possible. TLM procedures were classified as recommended by the European Laryngological Society (14, 15). Patients treated by TLM in a salvage setting and resulting exposed laryngeal cartilage were administered antibiotics (moxifloxacin 400 mg daily) during at least 10 days to prevent development of chondroradionecrosis (16). Following surgical treatment and pathologic examination of the resection specimen, the patients were re-discussed during the multidisciplinary tumor board meeting before any adjuvant therapy (second-look procedure or RT) was proposed. Second-look procedures were reserved for patients with positive deep section margins and/or multiple positive superficial margins. Negative margins were defined as those with >1 mm between margin and tumor, close margins were ≤1 mm from the tumor, and positive margins were margins with overt tumoral infiltration. Adjuvant RT was indicated in cases of massive tumoral infiltration of section margins combined with the surgeon’s persuasion that no clear margins would be achieved with a second-look TLM procedure or in case of a second-look operation with definitive pathology showing again positive deep or superficial margins (no such case was encountered however—see below). After termination of treatment, regular follow-up visits with rigid or flexible laryngoscopy were organized every 2 months during the first 2 years, every 3 months during the third year, every 4 months during the fourth year, and every 6 months thereafter. Baseline imaging of the neck (CT or MRI) was performed 3–4 months after treatment and was repeated 1 and 2 years after treatment to exclude locoregional recurrence in more advanced cases (pT2–pT3) (17). Yearly imaging of the chest (plain chest radiograph or CT chest) was performed to detect second primary pulmonary cancer.

### Data and Statistical Analysis

The data related to patient, tumor and treatment characteristics, and oncologic and functional outcomes were retrieved anonymously from the patient’s files. Information was collected on gender, ethyl and smoking history, previous treatment for head/neck malignancies, previous treatment for glottic malignancies, cTNM classification, tumor extension [subglottic extension, supraglottic extension, involvement of anterior commissure (AC), and involvement of posterior commissure], type of cordectomy, laryngeal resection margins, tumor histology, pT classification, tubefeeding following TLM, tracheotomy following TLM, hospitalization duration, complications, adjuvant treatment, length of follow-up, tumor recurrence, glottic second primary occurrence, laryngeal preservation rate, and cause of death. Salvage setting TLM is defined as TLM procedures carried out for glottic SCC after previous RT in the head and neck region (glottic and non-glottic with inclusion of the glottis area in the irradiation field). According to the criteria of Warren and Gates and its modification by the National Cancer Institute, second primary SCC of the glottis was defined as a metachronous glottic SCC developing later than 60 months after the index diagnosis (18–20). As such, local recurrence after TLM was defined as malignancy in the same laryngeal subsite as the primary tumor diagnosed within 60 months after the diagnosis of the first glottic malignancy. Tumors developed later were considered glottic second primaries resulting from field cancerization. Data were statistically analyzed using SPSS version 22.0 statistical software (IBM Corp., Armonk, NY, USA). Laryngeal preservation rate at end of follow-up and local control rate at end of follow-up were compared between primary and salvage TLM patients using the chi-square test. Kaplan–Meier methods were used to estimate ultimate local control with laser alone, laryngeal preservation rate, overall survival (OS), disease-free survival (DFS), disease-specific survival (DSS), and local recurrence-free survival (RFS). The endpoint for DFS was the date of the first recurrence (local, regional, or distant). The endpoint for local RFS was the date of diagnosis of a first local recurrence. Univariate analysis using Log-Rank testing was employed to compare these data between different subgroups. Statistical significance was defined at the *p* < 0.05 level.

## Results

### Patient and Treatment Characteristics

142 patients were included, 133 (93.7%) male and 9 (6.3%) female patients. Mean patient age at time of TLM was 68 years (range 35–89 years, SD 11.3 years). TLM was performed as a primary or up-front treatment in 109 (76.2%) cases whereas 33 (23.2%) patients were treated in a salvage setting for recurrent glottic cancer after prior irradiation (*n* = 28, 84.8% of salvage cases) or for second primary cancer of the glottis after prior (chemo)radiation for a non-glottic head and neck cancer (*n* = 5, 15.2% of salvage cases). No neck dissections were performed in accordance with current practice in treatment of early glottic SCC. Types of cordectomies [according to ELS classification (14, 15)] performed were as follows: type I (*n* = 46, 32.4%), type II (*n* = 21, 14.8%), type III (*n* = 41, 28.9%), type IV (*n* = 7, 4.9%), type Va (*n* = 14, 9.9%), type Vb (*n* = 1, 0.7%), type Vc (*n* = 3, 2.1%), type Vd (*n* = 1, 0.7%), and type VI (*n* = 5, 3.5%). Data about type of cordectomy performed were missing in three cases (2.1%). Mean hospital stay (including day of the operation) was 1.5 days (range 1–8 days, SD 0.8 days). No patients received a tracheotomy or a feeding tube. Only four patients (2.8%) experienced mild complaints of aspiration, which was managed by temporarily thickening of fluids. One primarily treated patient developed chondronecrosis which was managed conservatively. After TLM, 109 patients (76.2%) were submitted to a wait and see policy, with radiological follow-up in cases who proved peroperatively to be T2 or T3 or in case of any clinical doubt. Twenty-eight patients (19.7%) were scheduled for a second-look TLM procedure with re-resection because of a compromised deep margin or multiple superficial margins positive for SCC with an invasive component. Of interest, not a single second-look TLM procedure yielded residual malignancy after pathological examination. In five cases (3.5%), second-look TLM was renounced by the treating surgeon due to the expected low probability of radicality in a voice preserving setting, and RT was preferred after multidisciplinary discussion.

### Tumor Characteristics

Based on preoperative clinical and radiological findings, cT classification in the up-front TLM group was cT1a in 72 (66.1%), cT1b in 11 (10.1%), and cT2 in 26 (22.9%) patients. In the salvage group, patients were cT/rT classified as cT1a–rT1a in 17 (51.5%), cT1b–rT1b in 1 (3.0%), cT2–rT2 in 14 (42.4%), and cT3–rT3 in 1 (3.0%) patients. All patients were cN0. Retrospective review revealed that, due to the absence of a standardized registration protocol, accurate description of tumor extent as observed during the TLM procedure was often lacking. Data on absence or presence of subglottic extension, supraglottic extension and involvement of the anterior and posterior commissure were lacking in 96 (67.6%), 102 (71.8%), 67 (47.2%), and 65 (45.8%) cases, respectively. Of the patients with detailed data on tumor extension available, involvement of the aforementioned laryngeal subregions was present in 41.3, 32.5, 57.3, and 6.5%, respectively. After resection, the pathologist judged the margins as clear in 30 patients (21.1%), close in 9 patients (6.3%), positive in 50 cases (35.2%), and non-evaluable (due to laser coagulation artifacts and/or problems in orienting) in 53 patients (37.3%). Patients were definitively classified as carcinoma *in situ* (CIS) (*N* = 35, 24.6%), pT1a/rpT1a (*N* = 69; 48.6%), pT1b/rpT1b (*N* = 6; 4.2%), and pT2/rpT2 (*N* = 32; 22.5%).

### Oncologic Outcome and Survival

Mean follow-up for the overall population was 51.6 months (range 0–187 months, SD 38.4 months). Death occurred during follow-up in 32 patients (22.5% of the total population). In the up-front TLM group, 21 deaths occurred with 2 deaths (9.1% of deaths in this subgroup) being related to the glottic cancer, 1 death due to distant disease and the other related to progression of a third recurrence with the patient opting for a palliative setting. In the salvage TLM group, 2 out of 11 reported deaths was disease related (18.2% of deaths in this subgroup). In the upfront TLM group, 15 patients (13.8%) developed a local recurrence, 2 (1.8%) a regional recurrence, 1 (0.9%) a locoregional recurrence, and 1 patient (0.9%) developed distant disease. Four primarily treated patients (3.7%) developed a glottic second primary malignancy (time between diagnosis of the primary glottic cancer and the second primary glottic cancer >60 months). Patients with local recurrence were salvaged with a redo TLM procedure (*n* = 8), external beam irradiation of the larynx (*n* = 4), or combined TLM and RT (*n* = 3). Patients with second primary glottic cancer were salvaged with TLM (*n* = 2), irradiation (*n* = 1), or combined treatment (*n* = 1). Eventually, one total laryngectomy was performed during the course of disease in the up-front group in a patient who suffered from serious aspiration after having been irradiated for second primary glottic cancer. In the salvage group, 12 patients (36.4%) developed local recurrence of whom 3 were again salvaged with redo TLM and 8 with a total laryngectomy. Mean time interval between treatment and diagnosis of recurrence was 25 months (SD 16.5 months) in the primary group and 23.9 months (SD 17.9 months) in the salvage group (one-way ANOVA, *p* = 0.864). The difference in probability of local recurrence during follow-up between both patient groups proved statistically significant (chi-square; *p* = 0.004).

In a first analysis including second primary glottic cancers (see above: occurring >60 months following primary treatment) as failure, local control at the end of follow-up after one TLM treatment was 75.0% in the total population; this outcome was better (trend, not significant) in the up-front group (control rate of 78.6%) than in the salvage group (control rate of 63.6%) (chi-square, *p* = 0.083). This local control rate at the end of follow-up rose to 83.8% after eventual additional laser procedures (“ultimate local control rate with laser alone”). This local control rate with laser alone proved significantly better in the up-front TLM group (87.4%) when compared with the salvage group (72.7%) (chi-square, *p* = 0.047).

In a second analysis, looking at local control rates calculated by only including real local recurrences and excluding glottic second primaries, local control rate after one session of TLM was 80.3% in the total population (85.6% in primary group and 63.6% in salvage group, *p* = 0.006), and ultimate local control with laser alone (including eventual additional laser procedures) was 87.6% in the total population (92.3% in primary group and 72.7% in salvage group, *p* = 0.003).

**Figure 1:**
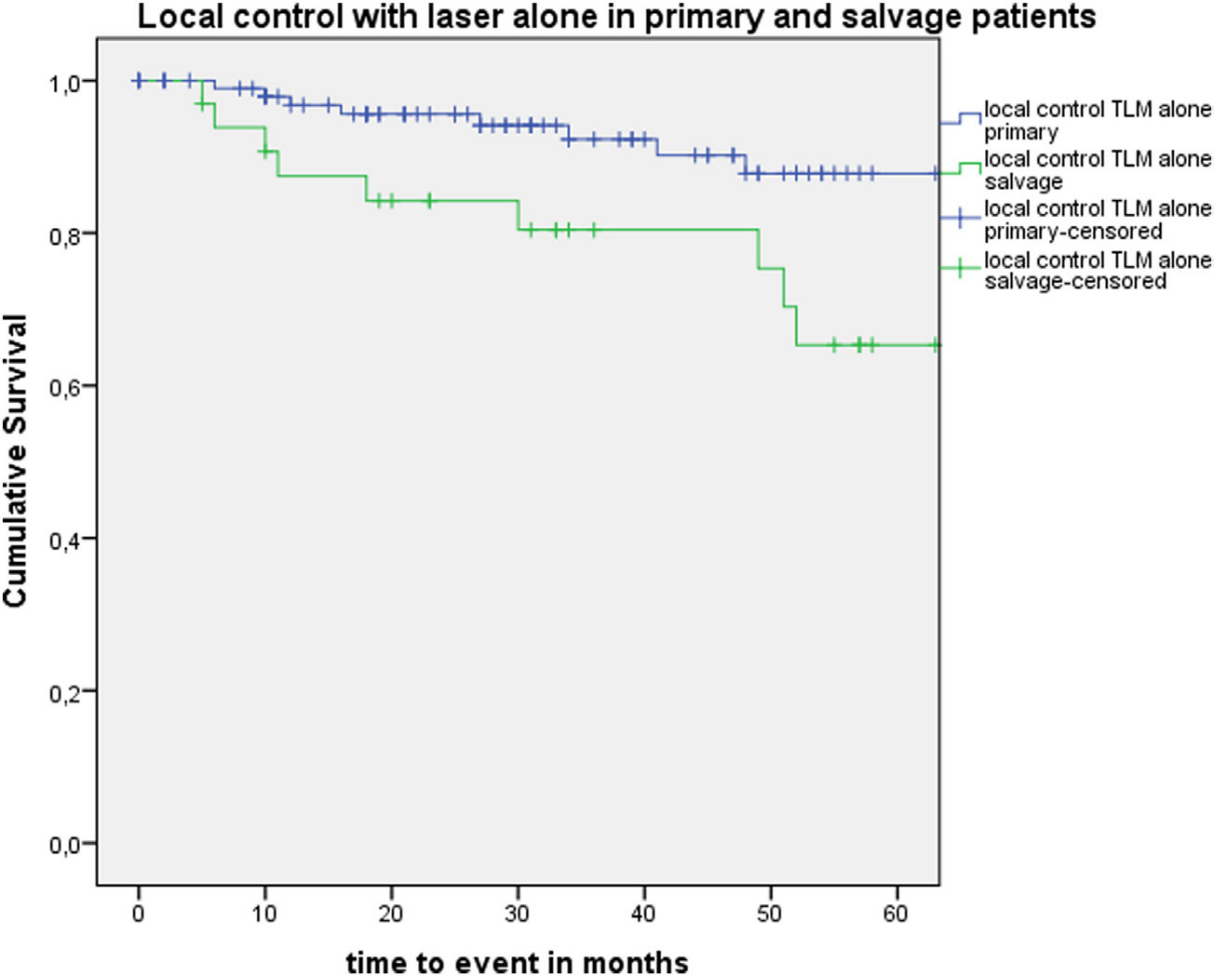
Kaplan–Meier curve illustrating local control with laser alone in patients treated with up-front or primary transoral laser microsurgery (TLM) (blue) and salvage TLM (green). Local control with laser alone was significantly higher in the primary group when compared with the salvage group (Log-Rank test, *p* = 0.012).

**Figure 2:**
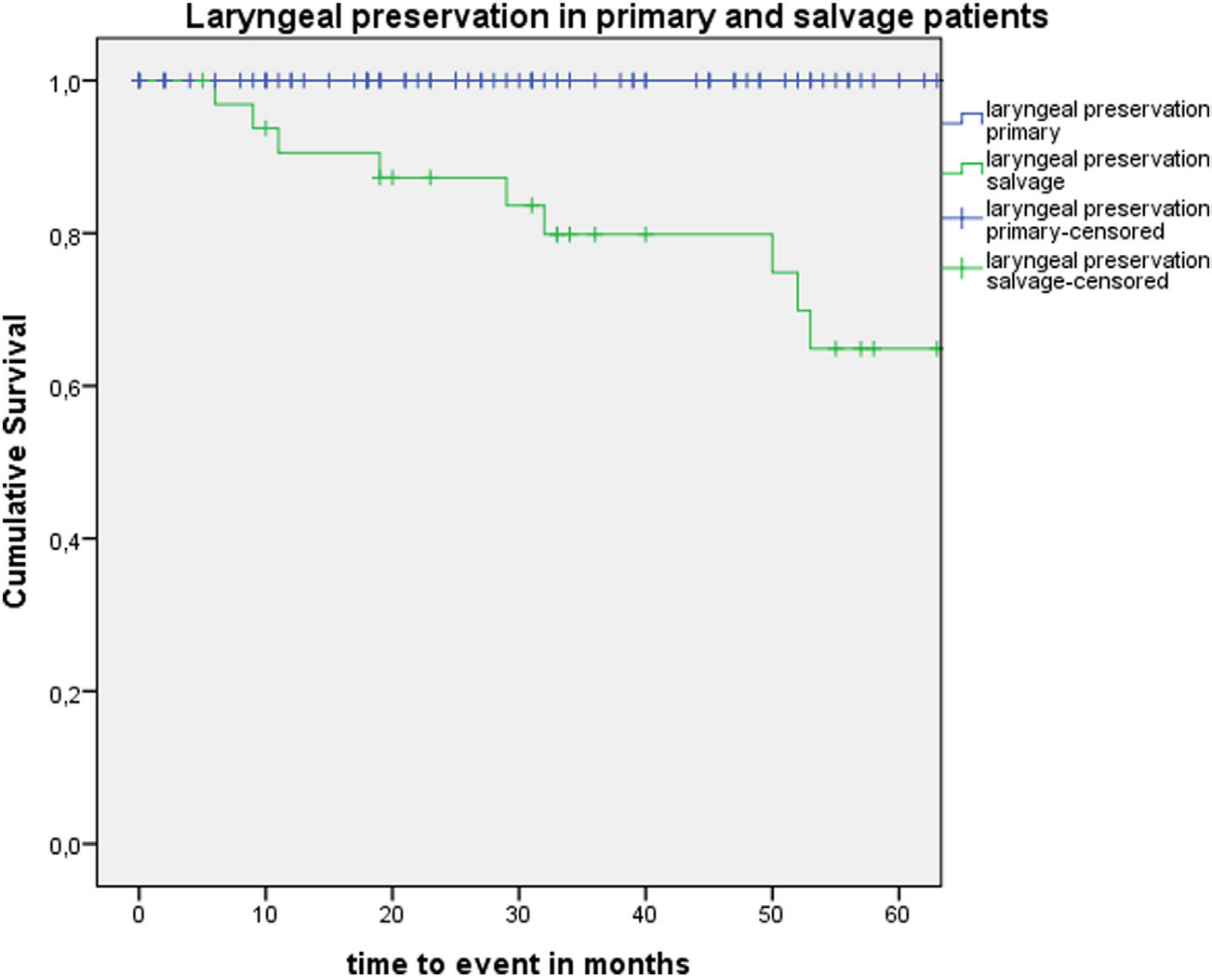
Kaplan–Meier curve illustrating laryngeal preservation in patients treated with up-front or primary transoral laser microsurgery (TLM) (blue) and salvage TLM (green). Laryngeal preservation was significantly higher in the primary group when compared with the salvage group (Log-Rank test, *p* < 0.001).

**Figure 3:**
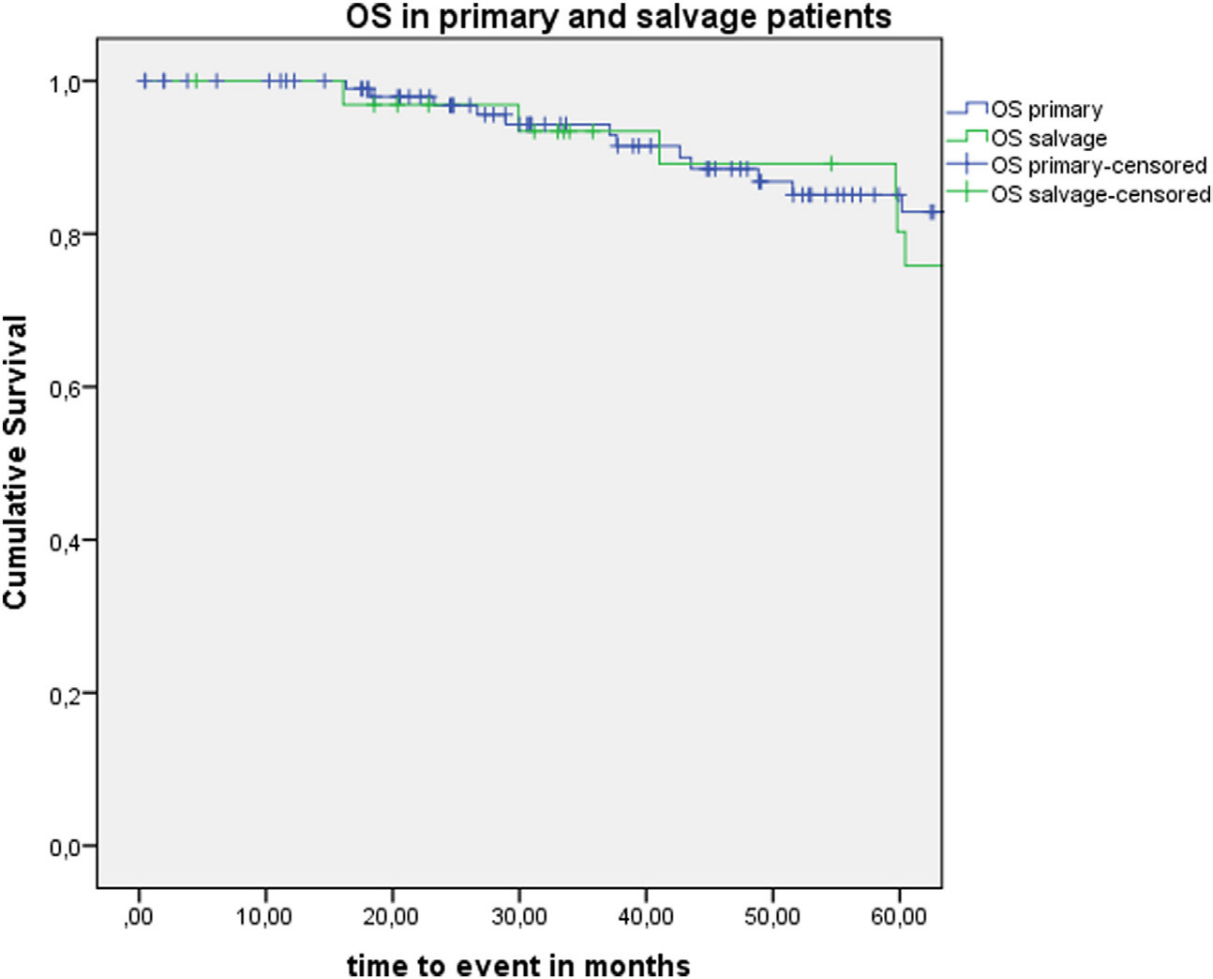
Kaplan–Meier curve illustrating overall survival (OS) in patients treated with up-front or primary transoral laser microsurgery (TLM) (blue) and salvage TLM (green). No difference in OS is observed between the primary and salvage group (Log-Rank test, *p* = 0.306).

**Figure 4:**
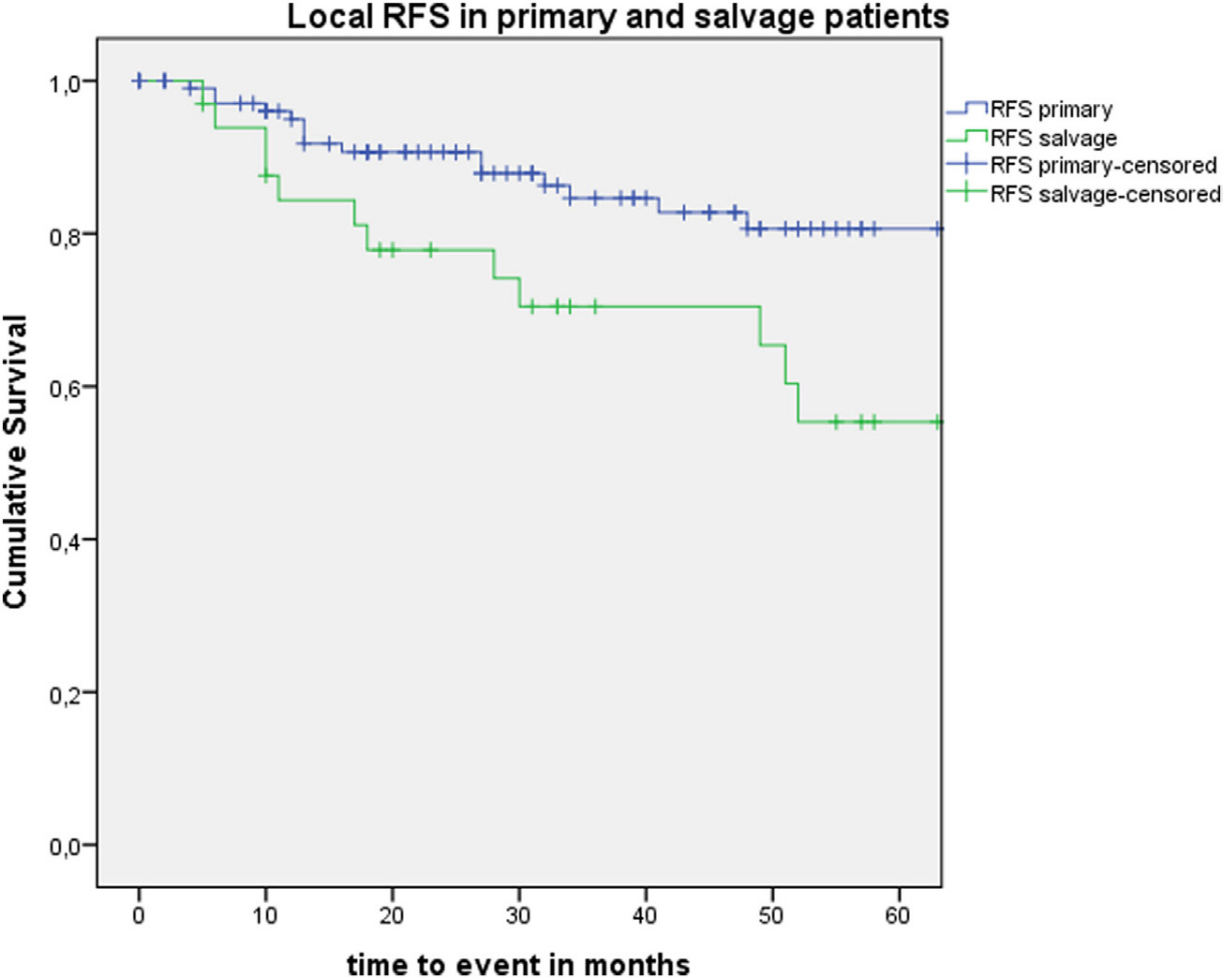
Kaplan–Meier curve illustrating local recurrence-free survival (RFS) in patients treated with up-front or primary transoral laser microsurgery (TLM) (blue) and salvage TLM (green). Local RFS was significantly higher in the primary group when compared with the salvage group (Log-Rank test, *p* = 0.014).

Five-year local control rate with laser alone (Kaplan–Meier), excluding glottic second primaries, was 80.9% (SE = 4.5%) in the overall group and proved significantly higher in the primary TLM group (87.8%, SE = 4.3%) compared with the salvage TLM group (65.3%, SE = 9.8%) (log rank, *p* = 0.012) (Figure 1). Kaplan–Meier estimated 5-year laryngeal preservation rate was 100% in the up-front group and 64.9% (SE = 9.8%) in the salvage group, a difference that proved statistically significant (Log-Rank, *p* < 0.001) (Figure 2). Looking in particular at patients who underwent a second-look procedure (*n* = 28, 6 salvage cases and 22 primary cases), 5 developed a local recurrence (3 primary cases and 2 salvage cases) of whom the 2 salvage cases were salvaged with a total laryngectomy. In the total patient group, 3-year OS was 94.1% (SE = 2.2%), 3-year DSS 100%, 3-year DFS 80.1% (SE = 3.8%), and 3-year local RFS was 81.0% (SE = 3.9%). Three-year OS in the up-front treated patient group was 94.3% (SE = 2.5%) while the 3-year OS in the salvage group was 93.4% (SE = 4.5%) (Log-Rank test, *p* = 0.306) (Figure 3). Three-year DSS was 100% both in the up-front TLM and salvage patient group (Log-Rank test, *p* = 0.298). Three-year DFS was 83.4% (SE = 4.1%) in the primary treated group and 70.4% (SE = 8.3%) in the salvage group (Log-Rank test, *p* = 0.061). Three-year local RFS was 84.6% (SE = 4.0%) after primary TLM and 70.4% (SE = 8.3) after salvage TLM (Log-Rank test, *p* = 0.014). As such, local RFS was significantly higher in the primary group when compared with the salvage group (Figure 4). Three- and five-year survival rates, laryngeal preservation rates, and local control rates with laser alone are summarized in Table 1.

**Table 1:** 3- and 5-year local control with laser alone, laryngeal preservation, and survival estimates (Kaplan–Meier) in the total population, the primary TLM group, and the salvage TLM group.

	3 years	5 years
Local control laser alone total population	89.2% (SE = 3.0%)	80.9% (SE = 4.5%)
Local control laser alone primary	92.3% (SE = 3.1%)	87.8% (SE = 4.3%)
Local control laser alone salvage	80.4% (SE = 7.2%)	65.3% (SE = 9.8%)
Laryngeal preservation total population	94.6% (SE = 2.2%)	89.7% (SE = 3.5%)
Laryngeal preservation primary	100%	100%
Laryngeal preservation salvage	79.8% (SE = 7.4%)	64.9% (SE = 9.8%)
OS total population	94.1% (SE = 2.2%)	83.3% (SE = 3.9%)
OS primary	94.3% (SE = 2.5%)	85.1% (SE = 4.2%)
OS salvage	93.4% (SE = 4.5%)	80.3% (SE = 8.0%)
DSS total population	100.0%	97.6% (SE = 1.7%)
DSS primary	100.0%	98.4% (SE = 1.6%)
DSS salvage	100.0%	95.5% (SE = 4.4%)
DFS total population	80.1% (SE = 3.8%)	68.1% (SE = 5.1%)
DFS primary	83.4% (SE = 4.1%)	72.9% (SE = 5.8%)
DFS salvage	70.4% (SE = 8.3%)	55.3% (SE = 10.1%)
RFS total population	81.0% (SE = 3.7%)	73.4% (SE = 4.7%)
RFS primary	84.6% (SE = 4.0%)	80.6% (SE = 4.7%)
RFS salvage	70.4% (SE = 8.3%)	55.3% (SE = 10.1%)

*DFS, disease-free survival; DSS, disease-specific survival; OS, overall survival; RFS, recurrence-free survival; TLM, transoral laser microsurgery*.

When the salvage group was restricted to patients with a local recurrence after previous RT for a glottic cancer (*n* = 28), thus excluding those patients with TLM for glottic carcinoma after having been irradiated for a non-glottic head and neck cancer (*n* = 5), the oncologic results in the salvage group did not change significantly when compared with the total salvage group (*n* = 33) upon Log-Rank analysis (DFS: *p* = 0.987, OS: *p* = 0.983, DSS: *p* = 0.929, laryngeal preservation rate: *p* = 0.796, local control rate with laser alone: *p* = 0.870).

**Figure 5:**
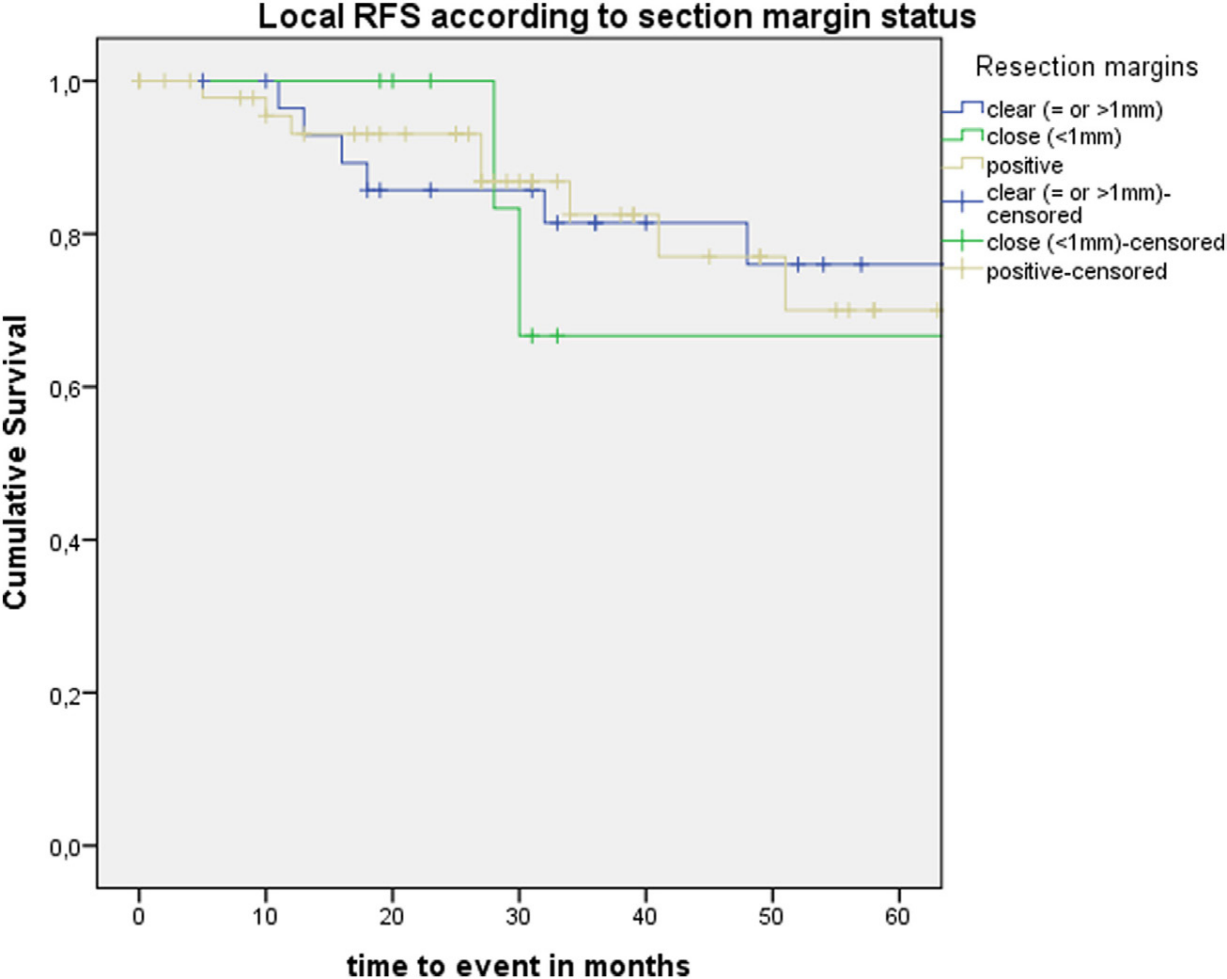
Kaplan–Meier curve illustrating local recurrence-free survival (RFS) according to section margin status.

**Figure 6:**
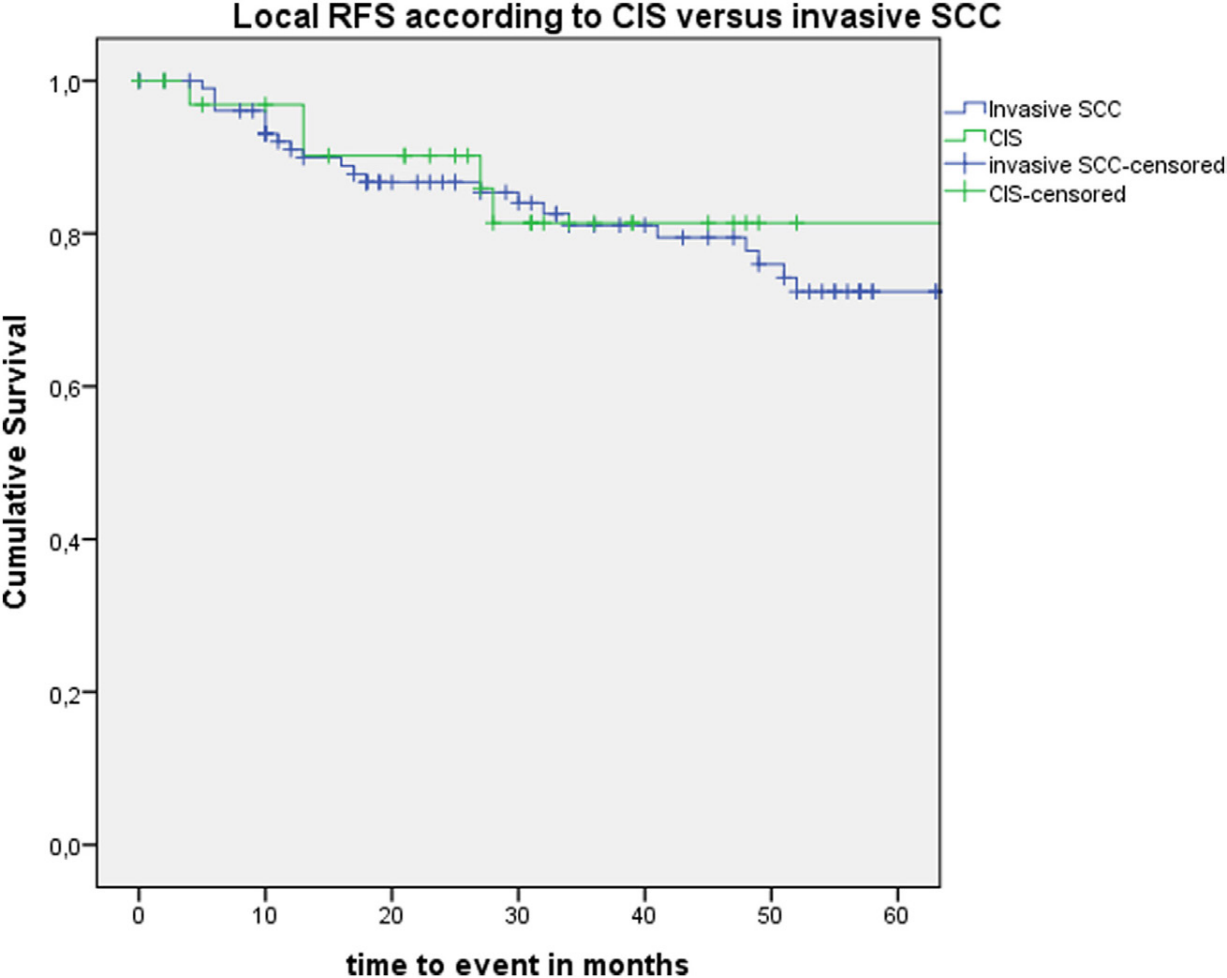
Kaplan–Meier curve illustrating local recurrence-free survival (RFS) according to presence of carcinoma *in situ* (CIS) or invasive squamous cell carcinoma (SCC).

After further univariate analysis, section margin status, cT classification, involvement of the AC, and subglottic extension did not seem to influence local control with laser alone, DFS, or local RFS. Surprisingly, no significant higher local control with laser alone, DFS, or local RFS were observed in patients with CIS upon definitive pathological examination when compared with invasive SCC. Results of these subgroup analyses are illustrated in Table 2 and Figures 5 and 6.

**Table 2:** *p*-Values after comparison of local control with laser alone, DFS, and local RFS between different subgroups using Log-Rank test.

Univariate analysis (Log-Rank)	Local control with laser alone	DFS	Local RFS
cT classification	*p* = 0.696	*p* = 0.690	*p* = 0.726
pT classification	*p* = 0.302	*p* = 0.628	*p* = 0.583
Subglottic extension	*p* = 0.244	*p* = 0.540	*p* = 0.927
Involvement of AC	*p* = 0.588	*p* = 0.288	*p* = 0.414
CIS versus invasive SCC	*p* = 0.103	*p* = 0.530	*p* = 0.715
Section margin status	*p* = 0.663	*p* = 0.961	*p* = 0.927

*AC, anterior commissure; CIS, carcinoma in situ; SCC, squamous cell carcinoma; RFS, recurrence-free survival; DFS, disease-free survival*.

## Discussion

Since the 1980s, TLM emerged as a surgical alternative to RT for the primary management of early glottic SCC and has nowadays a proven track record in the primary management of glottic cancer, combining high local control with excellent laryngeal preservation rates (1, 5–9, 21). In this single-center retrospective case series, we report on the oncologic outcomes of our 142 first TLM procedures, hence this includes patients at the beginning of the learning curve and also including primary cases as well as salvage cases. As expected in a population of patients with early glottic carcinoma, 3- and 5-year DSS and OS proved to be excellent in our series, both in the up-front TLM group and in the salvage TLM group. Local recurrence was observed with high predominance over regional recurrence or distant disease, which is another logical finding in this patient group. Three- and five-year ultimate local control rates (excluding glottic second primary cancers) obtained by “laser only treatment” (1 or successive TLM procedures) were satisfying and proved significantly better in the up-front TLM group (92.3% at 3 years and 87.8% at 5 years) when compared with the salvage group (80.4% at 3 years and 65.3% at 5 years) (Log-Rank; *p* = 0.012). However, mainly due to the adjuvant use of RT in patients who could not be cured by TLM procedures alone, 3- and 5-year laryngeal preservation rates in the up-front TLM group raised to an excellent 100 versus 79.8% (3 years) and 64.9% (5 years) in the salvage group (Log-Rank, *p* < 0.001). This excellent laryngeal preservation rate in primarily treated patients is comparable to the rates described in the literature (6, 7, 9, 21, 22) (ranging between 95 and 97.1%) and clearly illustrates the long-term benefit with regards to laryngeal preservation of primary TLM treatment, leaving all salvage options open, including RT, should the tumor recur. In this aspect, TLM and RT are not mere alternatives to each other but are complimentary treatments in selected patients, leading to optimal oncologic outcomes. This also stresses the importance of a multidisciplinary approach of the patient with early glottic cancer. Looking at oncologic results of primary TLM reported in the literature, our 5-year ultimate local control rate with laser alone (87.8%) is comparable to the ultimate local control rate with laser alone as reported by Peretti et al. (6) (92.7%). Our 5-year DFS is 72.9%, which is rather low when compared with series by Peretti et al. (6) (81.3%) and Ansarin et al. (7) (85.3%). However, the overall incidence of local recurrences during follow-up in primary treated patients (13.8%) is comparable to the incidence reported by Ansarin et al. (7) (12.2%) but lower than reported by Schrijvers et al. (21) (27%). Our estimated 5-year laryngeal preservation rate (100%) and 5-year DSS (98.4%) are among the best described in the literature. The dichotomy between a lower ultimate local control rate with laser alone on one hand and excellent DSS and laryngeal preservation rate on the other hand is due to a higher tendency in our centre to administer RT, either as an adjuvant treatment in patients with obvious section margin involvement or as a salvage option in cases of recurrence after primary TLM, when compared with other centers with high local control rates with laser alone, where redo laser procedures are preferred in these instances. The contrast between lower DFS but very high laryngeal preservation rate is a good illustration of the effectiveness of additional laser procedures and/or RT in saving the larynx in case of local recurrence.

Of particular interest in this series is the high amount of patients treated by TLM in a salvage setting (*N* = 33 or 23.2% of the total population). In the literature, the evidence on TLM as a salvage treatment for radiorecurrent laryngeal cancer is scarce, the series being retrospective and including small numbers of highly selected patients (16, 23–32). When compared with these reports, our series report on a substantial number of patients with radiorecurrent glottic cancer. A recent literature review on salvage TLM including all relevant literature concluded to local control rates with TLM alone varying between 50 and 87%, with a weighted average of 67%. The rates of definite laryngeal preservation, as obtained after TLM alone or a combination of TLM and salvage open partial laryngectomy, ranged from 50 to 94% with a weighted average of 73% (10). In our salvage group, 5-year local control with laser alone was 65.3%, and 5-year laryngeal preservation rate was 64.9%. Three-year estimates of OS in the literature range from 67.5 to 93.7% and 5-year estimates from 53 to 91%; for DSS, 3- and 5-year estimates both ranging between 68.6 and 100% are reported (10). In a systematic review, Ramakrishnan et al. calculated pooled mean estimates at 24 months for OS of 74.8% and for DFS of 70.9% (12). In our salvage patient series, 3- and 5-year OS were 93.4 and 80.3%, respectively; 3- and 5-year DSS were 100 and 95.5%, respectively; and 3- and 5-year DFS were 70.4 and 55.3%, respectively. These oncologic results are among the most favorable described in the literature and clearly illustrate the potential of salvage TLM to control the disease and avoid a total laryngectomy in well selected patients. Concerning resection margins after TLM for early glottic carcinoma, the relationship between section margin status and recurrence rate is unclear since a significant proportion of patients with reportedly involved margins never develop disease recurrence. In some patient series, close and positive margins were found to be independent risk factors for recurrence and poorer survival rates, while other studies were not able to confirm this negative impact of involved margin status (33). In an interesting recent study, Fiz et al. observed a reduction of DSS and RFS in pTis-T1b patients treated by TLM and positive multiple superficial and positive deep margins. In pT2 patients, DSS was reduced in positive multiple superficial margins, and RFS was reduced in positive single superficial, positive multiple superficial, and positive deep margins (34). In a review on TLM of early glottic cancer by Sjögren, rates of positive or inadequate margins between 24 and 51% were identified (22). In our series, margins were judged clear in 30 patients (21.1%), close in 9 patients (6.3%), positive in 50 cases (35.2%), and non-evaluable in 53 patients (37.3%). Second-look procedures were considered when multiple superficial margins and/or the deep margin were compromised, but apart from this pathologic margin status, the intraoperative opinion of the experienced surgeon on resection radicality seems the most important factor in decision-making. Eventually, a negative selection of 28 (19.7%) patients (where the surgeon had the most doubt on the radicality of the resection) was scheduled for a second-look TLM procedure with re-resection but not a single second-look TLM procedure yielded residual malignancy, suggesting a high rate of initially “false positive” involved section margins. This finding is supported by data from Ansarin et al. who observed persistence of disease in the resection specimens obtained by second-look TLM because of positive or close margins or dysplasia at margins in only 6 out of 90 patients (6.6%). Interestingly, they observed upon multivariate analysis that initial close to positive margins negatively influenced local recurrences, but also suggested that in this group with compromised margins, no difference in local recurrence rate was found between patients who underwent adjuvant RT or second-look procedure and those who underwent follow-up (7). Although these data need to be interpreted with caution due to small subpopulations and plausible selection bias with more aggressive tumors more likely to end up in the second-look or RT-arm, they put in doubt the added benefit of performing second-look procedures. However, until more robust data are available, is seems a sensible practice to perform second-look procedures for at least compromised deep margins. This pragmatic approach is supported by data from Peretti et al. who observed a low impact of superficial positive margins on local control with laser and organ preservation rates compared with deep infiltration or residual disease (6). The high proportion of margins considered by the pathologist as too difficult to assess and consequently too doubtful to make a reliable statement about is due to the small specimen sizes, laser coagulation and carbonization artifacts, tissue retraction, and orientation issues, which are well known particular problems after TLM. After having been confronted with this high rate of non-evaluable margins, our group searched for ways to counter these problems related to specimen orientation and evaluation. Inspired by earlier research in which a way to fix and orient surgical specimens on dehydrated cucumber was described (35, 36), we started to fix and meticulously orient the surgical samples on pig liver slices immediately after resection. The carrier-mounted specimens are sent to the pathologist, accompanied by a photograph of the tumor *in situ*. This new technique is assessed in an ongoing prospective trial and awaits objective validation. In our series, AC involvement could not be identified as a negative prognosticator. Although AC involvement has been found to be a risk factor for local recurrence (37), some large series could not confirm this negative impact on local control (6, 7). It seems appropriate to differentiate between tumors with AC involvement in the horizontal plane and those tumors in which the AC involvement extends in the vertical plane to the subglottic and/or supraglottic region. The latter tumor group carries a risk of poorer local control due to the close relationship with the underlying visceral spaces (pre-epiglottic space and subglottic area) (38). In radiorecurrent carcinoma, this anterior transcommissural extension was identified as a negative predictor on OS (39).

Evidently, our study has limitations. As a retrospective study, inherent selection bias cannot be excluded. Moreover, because of the small study population and small subgroups, multivariate analysis was not possible. As the primary TLM group and salvage TLM group are two completely different cohorts, statistical comparison needs to be interpreted with caution. However, a rudimentary statistical comparison of outcomes is of interest for the reader because it confirms some obvious differences between primary and salvage groups (e.g., lower probability of local recurrence during follow-up, better local control with laser alone, better laryngeal preservation rate, and higher local RFS in the primary group) but it also illustrates that other oncologic outcomes of the difficult-to-treat salvage group are not worse when compared with the primary group (OS, DSS, and RFS). This is an important finding, as indications of TLM are progressively stretched and salvage TLM is becoming more widespread. As such, the comparison of both groups only tries to give the reader an idea about how results of TLM in a salvage group relate to those in a primary group, which is useful information both for the surgeon as for the patient. During data retrieval, we observed high rates of lacking data concerning the detailed extension of the tumor as observed during the TLM procedure (supraglottic and subglottic extension, involvement of the anterior and posterior commissure), making subgroup analysis even more difficult. As a reaction to this finding, we are evolving to a systematic and standardized way of reporting visualized tumor extension during TLM, making more detailed future evaluations possible. Another drawback of our study is the lack of objective pre- and postoperative voice assessments. As complications after TLM procedures, both in the primary and salvage setting, are very rare, postoperative voice quality warrants most attention when addressing functional outcomes of TLM. In our study, no valuable statements could be made on this important functional aspect due to the lack of systematically performed pre- and postoperative vocal assessment. Finally, as mentioned earlier, a high rate of non-evaluable margins were encountered due to orientation and processing difficulties experienced by the pathologist.

## Conclusion

This single-center retrospective case series confirms excellent definitive oncologic outcomes of up-front TLM for early glottic cancer with a 5-year laryngeal preservation rate of 100% and 5-year DSS of 98.4%. In the salvage setting, TLM allows avoidance of total laryngectomy in 64.9% of cases after 5 years. Upon univariate analysis, local control rate with laser alone, laryngeal preservation rate, and local RFS were significantly higher in the up-front TLM group when compared with the salvage TLM group, while OS, DSS, and DFS did not show significant differences between the subgroups.

## Ethics Statement

This study was carried out in accordance with the recommendations of the Institutional Review Board (University Hospital Leuven). The protocol was approved by the Committee for Medical Ethics of the University Hospitals Leuven. Informed consent for retrospective studies with anonymized data is not required according to Belgian law.

## Author Contributions

JM: data quality control, data analysis (statistics), drafting manuscript, and review of manuscript. JB: data collection, data analysis (statistics), and drafting manuscript. PD: drafting manuscript and review of manuscript. VP: data quality control, drafting manuscript, and review of manuscript.

## Conflict of Interest Statement

The authors do not have any potential conflict of interest to declare in relation with the content of this article.
